# A 2-methoxyestradiol bis-sulphamoylated derivative induces apoptosis in breast cell lines

**DOI:** 10.1186/s13578-015-0010-5

**Published:** 2015-04-22

**Authors:** Michelle Helen Visagie, Lyn-Marie Birkholtz, Anna Margaretha Joubert

**Affiliations:** Department of Physiology, University of Pretoria, Private Bag X 323, Arcadia, 0007 South Africa; Department of Biochemistry, Centre for Sustainable Malaria Control, University of Pretoria, Private Bag X20, Hatfield, Pretoria 0028 South Africa

**Keywords:** EMBS, Apoptosis, xCELLigence, p53, Bcl-2, Caspase

## Abstract

**Introduction:**

Research involving antimitotic compounds identified 2-methoxyestradiol (2ME2), as a promising anticancer endogenous metabolite. Owing to its low bioavailability, several *in silico*-designed 2ME2 analogues were synthesized. Structure-activity relationship studies indicated that an already existing 17-β-estradiol analogue, namely (8*R*,13*S*,14*S*,17*S*)-2-ethyl-13-methyl-7,8,9,11,12,13,14,15,16,17-decahydro-6H-cyclopenta[a]phenanthrane-3,17-diyl bis(sulphamate) (EMBS) to exert potential in vitro anticancer activity.

**Methods:**

This study investigated the in vitro apoptotic influence of EMBS in an estrogen receptor-positive breast adenocarcinoma epithelial cell line (MCF-7); an estrogen receptor-negative breast epithelial cell line (MDA-MB-231) and a non-tumorigenic breast cell line (MCF-12A). Cell cycle progression, a phosphatidylserine flip, caspase 6-, 7- and 8 enzyme activity levels, Bcl-2 phosphorylation status at serine 70 and Bcl-2- and p53 protein levels were investigated to identify a possible action mechanism for apoptotic induction.

**Results:**

The xCELLigence real-time label-independent approach revealed that EMBS exerted antiproliferative activity in all three cell lines after 24 h of exposure. A G_2_M block was observed and apoptosis induction was verified by means of flow cytometry using propidium iodide and Annexin V-FITC respectively. EMBS-treated cells demonstrated a reduced mitochondrial membrane potential. EMBS exposure resulted in a statistically significant increase in p53 protein expression, decreased Bcl-2 protein expression and a decrease in pBcl-2(s70) phosphorylation status in all three cell lines. Results support the notion that EMBS induces apoptosis in all three cell lines.

**Conclusion:**

This study includes investigation into the apoptotic hallmarks exerted by EMBS after exposure of three cell lines namely MCF-7-, MDA-MDA-231- and MCF-12A cells. Increased caspase 6-, caspase 7- and caspase 8 activities, upregulation of p53 protein expression and a decrease in phosphorylation status of Bcl-2 at serine 70 in tumorigenic and non-tumorigenic lines were demonstrated.

## Introduction

For decades antimitotic agents have been studied for their potential anticancer activity. These studies have resulted in the discovery of 2-methoxyestradiol (2ME2), an endogenous 17β-estradiol that exerts antiproliferative, antiangiogenic, and anti-inflammatory activity in an estrogen receptor-independent manner [1]. 2ME2 has estrogen-independent activity that has stimulated substantial curiosity regarding its mechanism of action, including its relevance as a therapeutic agent [2].

Owing to its limited bioavailability, several estradiol analogues have been designed *in silico* and subsequently synthesized [3]. In structure-activity relationship studies it has been reported that the addition of sulphamate groups (position 3 and 17) to 2ME2 results in superior efficacy and bioavailability [2]. This improvement is due to the sulphamate groups inhibiting activation of metabolism and deactivation of conjugation and interaction with carbonic anhydrase [4]. The sulphamate group reversibly interacts with the catalytic site of carbonic anhydrase II, an enzyme highly expressed in red blood cells, making it possible for the sulphamoylated compound to bypass first pass liver metabolism [2]. The superior bioavailability is supported by a bis-sulphamoylated analogue (position 3 and 17), 2-methoxyestradiol-bis-sulphamate, which exerts antiproliferative activity in several cell lines including a lung adenocarcinoma cell line (HOP-62), a human colorectal carcinoma cell line (HCT-116), human glioblastoma cells (SF-539), a melanoma cell line (UAVC-62), a human ovarian adenocarcinoma cell line (OVCAR-3), renal carcinoma cells (SN12-C), human prostate carcinoma cells (DU-145) and a breast tumorigenic cell line (MDA-MB-435) [5]. In addition, 2-methoxyestradiol-bis-sulphamate is resistant to in vivo metabolisms in xenografts derived from human melanoma (MDA-MB-435) cells. Superior activity is demonstrated by 85% bioavailability and maintained anticancer activity 28 days after treatment cessation [5].

The addition of methyl and ethyl groups to the estradiol results in antiproliferative and antimitotic activity independent of the estrogen receptors. The antimitotic ability of these compounds is due to their ability to bind the colchicine binding-site of tubulin [6]. The latter is supported as estradiol compounds with ethyl additions at position 2 (2-ethyl-3-*O*-sulphamoyl-estra-1,3,5(10)16-tetraene and 2-ethyl-3-*O*-sulphamoyl-estra-1,3,5(10)15-tetraen-17-ol) exerting antimitotic activity in non-tumorigenic MCF-12A, tumorigenic MCF-7 and metastatic MDA-MB-231 breast cancer cells. In addition, both these derivatives were found to inhibit carbonic anhydrase II activity and mimic carbonic anhydrase IX activity in MDA-MB-231 cells. Carbonic anhydrase II is not overexpressed in several types of cancer contributing to the acidic environment found in the tumours [7,8].

This study involved an estradiol compound that was modified by the addition of an ethyl group (position 2), a methyl group (position 3) and bis-sulphamoylation at positions 3 and 17 [4]. These chemical modifications merit investigation of possible in vitro anticancer activity exerted by (8*R*,13*S*,14*S*,17*S*)-2-ethyl-13-methyl-7,8,9,11,12,13,14,15,16,17-decahydro-6H-cyclopenta[a]phenanthrane-3,17-diyl bis(sulphamate) (EMBS). The study investigated the influence of EMBS on proliferation, cell cycle progression, caspase activation and subsequent apoptosis induction in an estrogen receptor-positive breast adenocarcinoma cell line (MCF-7), a triple negative metastatic breast cell line (MDA-MB-231) and a non-tumorigenic breast epithelial cell line (MCF12A).

## Materials and methods

### Cell lines

The MCF-7 cell line is an estrogen receptor-positive tumorigenic adherent breast epithelial cell line derived from metastatic sites in adenocarcinoma [9]. MCF-7 cells were supplied by Highveld Biological (Pty) Ltd. (Sandringham, Gauteng, South Africa).

The MDA-MB-231 cell line is an estrogen receptor-negative tumorigenic metastatic breast cell line and is commercially available from Microsep (Pty) Ltd. (Johannesburg, Gauteng, South Africa) [9]. Tumorigenic cells were grown and maintained in sterile 25 cm^2^ tissue culture flasks at a humidified atmosphere at 37°C and 5% CO_2._ Growth medium consisted of Dulbecco’s Minimum Essential Medium Eagle (DMEM) supplemented with 10% heat-inactivated fetal calf serum (56°C, 30 min), 100 U/ml penicillin G, 100 μg/ml streptomycin and fungizone (250 μg/l).

MCF-12A cells are non-tumorigenic transformed adherent human breast epithelial cells. These cells are produced by long-term cultures and form domes in confluent cultures [9]. The MCF-12A cells were a gift from Prof Parker (Department of Medical Biochemistry, University of Cape Town, Western Cape, South Africa). MCF-12A cells were cultured in growth medium consisting of a 1:1 mixture of DMEM and Ham’s-F12 medium, 20 ng/ml epidermal growth factor, 100 ng/ml cholera toxin, 10 μg/ml insulin and 500 ng/ml hydrocortisone, supplemented with 10% heat-inactivated fetal calf serum (56°C, 30 min), 100 U/ml penicillin G, 100 μg/ml streptomycin and fungizone (250 μg/l).

### Reagents

All required reagents of cell culture analytical grade were purchased from Sigma (St. Louis, MI, United States of America) unless otherwise specified. Heat-inactivated fetal calf serum (FCS), sterile cell culture flasks and plates were purchased from Sterilab Services (Kempton Park, Johannesburg, Gauteng, South Africa). Penicillin, streptomycin and fungizone were obtained from Highveld Biological (Pty) Ltd. (Sandringham, Gauteng, South Africa). The Annexin V fluorescein isothiocyanate (FITC) kit, Mitocapture Mitochondrial Apoptosis Detection Kit, Caspase 6 colorimetric assay kit, the Flice/Caspase 8 colorimetric assay, Rabbit polyclonal antibody for anti-active caspase 7, the anti-rabbit antibody conjugated to Dylight^™^ 488, ImmunoSet p53/MDM2 Complex Elisa development set, RIPA cell lysis buffer, TMB substrate and Plates (ImmunoSet pack) were purchased from BIOCOM biotech (Pty) Ltd. (Clubview, Gauteng, South Africa). The Flow Collect Bcl-2 activation dual detection kit was purchased from Merck Millipore (Ltd) (Billerica, MA, United States of America).

EMBS is commercially unavailable and was synthesized by iThemba Pharmaceuticals (Pty) Ltd. (Modderfontein, Gauteng, South Africa) [3]. A stock solution of EMBS dissolved in dimethyl sulphoxide (DMSO) was prepared with a concentration of 10 mM and was stored at 4°C. The vehicle control sample composed of DMSO and growth medium and the DMSO content of the final dilutions never exceeded 0.05% (v/v). In addition, cells were exposed to actinomycin D (0.1 μg/ml; 24 h) as positive control for apoptosis.

## Methods

### Cell proliferation

The xCELLigence method is a system-based assay developed by Roche Applied Science (Penzberg, Germany) to demonstrate cell growth, adhesion and morphology in real-time, utilizing a label-independent manner and was employed to demonstrate the effects of EMBS on proliferation. This system measured electrical impedance across the micro-electrodes integrated on the bottom of tissue culture 96-well plates allowing real-time and continuous cellular analysis as cells attach and proliferate [10,11]. The change in impedance was expressed as the cell index that is an indication of cell number, cellular attachment and morphology. By plotting cell index values over time, a precise real-time cellular analysis profile was generated. The response of live cells to EMBS supplied proliferative and antiproliferative information.

The RTCA SP Station was connected to the RTCA control unit and the xCELLigence system was tested by resistor plate verification prior to the xCELLigence system being placed inside the incubator at a humidified atmosphere at 37°C and 5% CO_2._ Background quantification was done by adding 100 μl of the appropriate growth medium to the wells. Subsequently the plate was calibrated using RTCA software package 1.2. Cells were seeded at 5000 cells per well, placed on a rotator for 30 min at room temperature and cells were subsequently placed in the xCELLigence system linked to the incubator in a humidified atmosphere at 37°C and 5% CO_2_ [10,11]. The xCELLigence system monitored cell adhesion and proliferation for 24 h to allow for attachment. Subsequently, cells were exposed to 0.2-1 μM EMBS concentration series since previous studies demonstrated optimal antiproliferative activity after 0.4 μM EMBS exposure for 24 h [12]. As the cells multiplied, the electrical impedance created by the cells was converted to cell index values corresponding to each well. These cell index values are then directionally proportionate to cell number, size and attachment. Cell adhesion and proliferation were measured for the next 72 h.

### Cell cycle progression

Flow cytometry and propidium iodide was utilized to investigate the DNA content to determine cell cycle distribution, G_2_/M block and the presence of a sub-G_1_ apoptotic peak [13]. After 24 h of exposure to 0.4 μM EMBS, cells were trypsinized and resuspended in 1 ml growth medium. Cells (1 × 10^6^) were centrifuged for 5 min at 300x *g*. The pellet was resuspended twice in ice-cold phosphate buffer solution (PBS). The supernatant was discarded and cells were resuspended in 200 μl of ice-cold PBS containing 0.1% FCS. Ice-cold 70% ethanol (4 ml) was added in a drop-wise manner and cells were stored at 4°C for 24 h. After 24 h, cells were pelleted by centrifugation for 5 min. The supernatant was removed and cells were resuspended in 1 ml of PBS containing propidium iodide (40 μg/ml) and incubated at 37°C, 5% CO_2_ for 45 min. Cells were then analyzed by means of FACS FC500 System flow cytometer (Beckman Coulter South Africa (Pty) Ltd) equipped with an air-cooled argon laser excited at 488 nm. Data from at least 10 000 cells were captured with CXP software (Beckman Coulter South Africa (Pty) Ltd, Johannesburg, Gauteng, South Africa] and analyzed with Cyflogic (CyFlo Ltd., Turku, Finland).

### Apoptosis induction

The presence of apoptosis was evaluated and quantified using flow cytometry in combination with Annexin V-fluorescein isothiocyanate (FITC). In apoptosis, the calcium-dependent phospholipid scramblase activity is activated which results in the externalization of the phosphatidylserine layer of the cell membrane [14]. Externalization of the phosphatidylserine layer during apoptosis provides binding sites for Annexin V. Annexin V (a Ca^2+^-dependent, phospholipid binding protein) is conjugated to a fluorochrome; this allows for identification of early- and late apoptosis and necrosis [14]. After 24 h of exposure to 0.4 μM EMBS, cells were trypsinized and 10^6^ cells were resuspended in 1 ml of 1x Binding Buffer and centrifuged at 300x *g* for 10 min. Supernatant was removed and cells were resupended in 100 μl of 1*x* Binding Buffer. Annexin V-FITC (10 μl) was added and incubated for 15 min in the dark at room temperature. After 15 min, cells were washed by adding 1 ml of 1*x* Binding Buffer and centrifuged at 300x *g* for 10 min. Supernatant was carefully removed and cells were resuspended in 500 μl of 1*x* Binding Buffer solution. Immediately prior to analysis, 12.5 μl of propidium iodide (40 μg/ml) was added and samples were mixed gently. Propidium iodide fluorescence (oncotic cells) and annexin V fluorescence (apoptotic cells) were measured with FC500 System flow cytometer (Beckman Coulter South Africa (Pty)Ltd.) equipped with an air-cooled argon laser excited at 488 nm. Data from at least 30 000 cells were analyzed with CXP software (Beckman Coulter South Africa (Pty) Ltd). Propidium iodide emits light at 617 nm and FITC emits light at 530 nm. Data were obtained from the log forward scatter detector nr 1 (FL1 Lin, 515–545 nm emissions) and the log forward scatter detector nr 3 (FL3 Lin, 600 nm emissions) were represented as a single dot-plot. Distributions of cells within the quadrants were calculated with Cyflogic version 1.2.1 software (Pertu Therho, Turko, Finland).

### Mitochondrial membrane potential

Further studies were conducted that employed flow cytometry and Mitocapture Mitochondrial Apoptosis Detection Kit to demonstrate the apoptosis pathway utilized by EMBS. Mitochondrial integrity was investigated by means of a unique cationic dye – 5,5’,6,6’-tetrachloro-1,1’,3,3’- tetraethylbenzimidazolylcarbocyanine iodide [8]. Reduction of the mitochondrial membrane potential is an early feature of apoptosis, which is due to the loss of the electrochemical gradient across the mitochondrial membrane [15]. Cells (500 000) were seeded with an overnight attachment policy. After 24 h of exposure to 0.4 μM EMBS, cells were detached using trypsin and centrifuged at 13,000*x* g. Cells (500 000) were resuspended in 1 ml of diluted Mitocapture solution (1 μl mitocapture: 1 ml pre-warmed incubation buffer) incubated in a humidified atmosphere (37°C, 5% CO_2_). After 20 min, cells were centrifuged at 500x *g*, the supernatant discarded and cells resuspended in 1 ml of prewarmed incubation buffer (37°C). Cells were analyzed with a FC500 System flow cytometer (Beckman Coulter South Africa (Pty) Ltd.). Apoptotic cells were detected in the FITC channel (usually FL1) and showed diffused green fluorescence. Data from at least 10 000 cells were analyzed by means of Cyflogic version 1.2.1 software (Pertu Therho, Turko, Finland).

### Caspase activation

Possible activation of caspase 6 and −8 were investigated by means of caspase 6 and FLICE/caspase 8 colorimetric kits, respectively. Caspase 7 activation was demonstrated using flow cytometry and rabbit anti-active caspase 7. This allowed for verification of apoptosis induction as caspase 7 and −8 are executioner caspases and caspase 8 is an initiator caspase involved in the death receptor pathway [16].

### Caspase 6 and caspase 8

Cells (500 000) were seeded with an overnight attachment policy. Subsequently, cells were exposed to 0.4 μM EMBS for 24 h. Cells were trypsinized and centrifuged at 12 000 rpm. Subsequently 500 000 cells were resuspended in 50 μl of chilled cell lysis buffer and incubated on ice for 10 min. Cells were centrifuged at 10 000x *g* for 1 min and the supernatant incubated on ice. After the protein concentration had been determined with the use of the BCA protein assay (Thermo Fisher Scientific, Johannesburg, Gauteng, South Africa), 100 μg protein/50 μl cell lysis buffer was mixed with 50 μl 2*x* reaction buffer (containing 10 mM DTT). Afterwards, 5 μl 4 mM Ac-Leu-Glu-His-Asp-*p*-nitroanilide (Ac-VEID-*p*NA) (caspase-6-specific substrate), Ac-Ile-Glu-Thr-Asp-*p*-nitroanilide (Ac-IETD-*p*NA) (caspase-8-specific substrate) (200 μM final concentration) were added and incubated at 37°C for 120 min. Absorbances were determined at 405 nm on the EL_x_800 Universal Microplate Reader available from Bio-Tek Instruments Inc. (Winooski, VT, United States of America).

### Caspase 7

Cells were seeded at 500 000 cells per flask and after 24 h of attachment, cells were exposed to 0.4 μM EMBS for 24 h. Subsequently, cells were trypsinized and 500 000 cells were centrifuged and the medium was discarded. Cells were then resuspended in wash buffer and centrifuged. The supernatant was removed and cells were resuspended in fixation buffer (0.1% formaldehyde) and incubated for 20 min at room temperature. Afterwards, the supernatant was removed; cells were resuspended in assay buffer (1% bovine serum albumin) and centrifuged. Cells were resupended in 500 μl ice-cold permeabilization buffer (100% methanol) and put on ice for 10 min and subsequently washed twice in assay buffer. Cells were incubated with 15 μg/ml primary antibody (rabbit anti-active caspase 7) (100 μl) on ice for 90 min after which 900 μl assay buffer was added. After centrifugation samples were washed twice with 500 μl assay buffer, centrifuged and resuspended in 100 μl assay buffer with 0.2 μg/ml anti-rabbit antibody conjugated to Dylight^™^ 488 fluorochrome. Samples were incubated on ice for 60 min in the dark, 900 μl assay buffer added and washed as before. Fluorescence was then measured using the FL1 channel with a FC500 System flow cytometer equipped with an air-cooled argon laser excited at 488 nm (Beckman Coulter South Africa (Pty)Ltd). Data from at least 10 000 cells were analyzed with CXP software (Beckman Coulter South Africa (Pty) Ltd).

### Expression of p53

The tumor suppressor p53 protein is essential for genetic integrity and is mutated in over half of all human cancers [17]. The influence of EMBS on p53 protein expression was demonstrated with the use of spectrophotometry. Cells were seeded at a density of 5 000 cells per 96 wells and left for 24 h to allow for attachment. Cells were exposed to 0.4 μM EMBS for 24 h. After the exposure period, 100 μl assay buffer (mixed according to suppliers’ instructions) was added to the control wells. Standards (100 μl) and samples prepared in assay buffer were added to the wells. The plate was sealed and incubated for 1 h at room temperature. Wells were emptied and washed with 400 μl wash buffer thrice. The detection antibody (100 μl) (mixed according to suppliers instructions) was added to each sample, except to the blank, and the plate was sealed for 1 h. Wells were emptied and washed thrice with 400 μl wash buffer. TMB solution (100 μl) was pipetted into each well and the plate was sealed at room temperature. After 30 min, 1 N hydrochloric acid was added and absorbance was read at 450 nm using EL_x_800 Universal Microplate Reader (Bio-Tek Instruments Inc., Vermont, United States of America).

### Bcl-2 expression

Bcl-2 is an anti-apoptotic protein belonging to the B-cell lymphoma-2 (BCL-2) family. Bcl-2 opposes apoptosis by binding to the proapoptotic members and neutralizing their activity [18]. Flow cytometry demonstrated the in vitro effects of EMBS on Bcl-2 protein expression and Bcl-2 phosphorylation at position 70. Exponentially growing MCF-7, MDA-MB-231 and MCF-12A cells were seeded at 500 000 cells per 25 cm^2^ flask. After 24 h of attachment, the medium was discarded and cells were exposed to 0.4 μM EMBS for 24 h. Cells were trypsinized and washed with 1*x* wash buffer. Samples were centrifuged and fixation buffer (0.5 ml) was added to the samples and incubated at room temperature for 20 min. Samples were centrifuged for 3 min and resuspended in 0.5 ml 1 X assay buffer. After centrifugation (2500 rpm) samples were resuspended in ice-cold 1 X permeabilization buffer, left on ice for 10 min and centrifuged at 2500 rpm and resuspended in 0.5 ml assay buffer and centrifuged again. Samples were stained using 5 μl 20 X antibody (as described by suppliers) and incubated in the dark. After 60 min, 900 μl 1 X assay buffer was added to the samples and cells were centrifuged for 3 min (2500 rpm). Samples were resuspended in 1 ml 1 X assay buffer and analyzed using the FC500 System flow cytometer (Beckman Coulter South Africa (Pty) Ltd). Data from at least 10 000–30 000 cells were analyzed by means of Cyflogic version 1.2.1 software (Pertu Therho, Turko, Finland).

### Statistics

Data were obtained from 3 independent experiments. All methods conducted in 96-well plates were carried out in triplicate for each independent experiment. Fluorescent measurement was expressed as a ratio of the value measured for the EMBS-treated cells compared to vehicle-treated exposed cells (mean relative fluorescence). Flow cytometry analysis involved data from at least 10 000 events that were repeated thrice, after which a representative figure was chosen for each experiment. Flow cytometry data were analyzed by means of Cyflogic version 1.2.1 software (Pertu Therho, Turko, Finland).

## Results

### Cell proliferation

**Figure 1:**
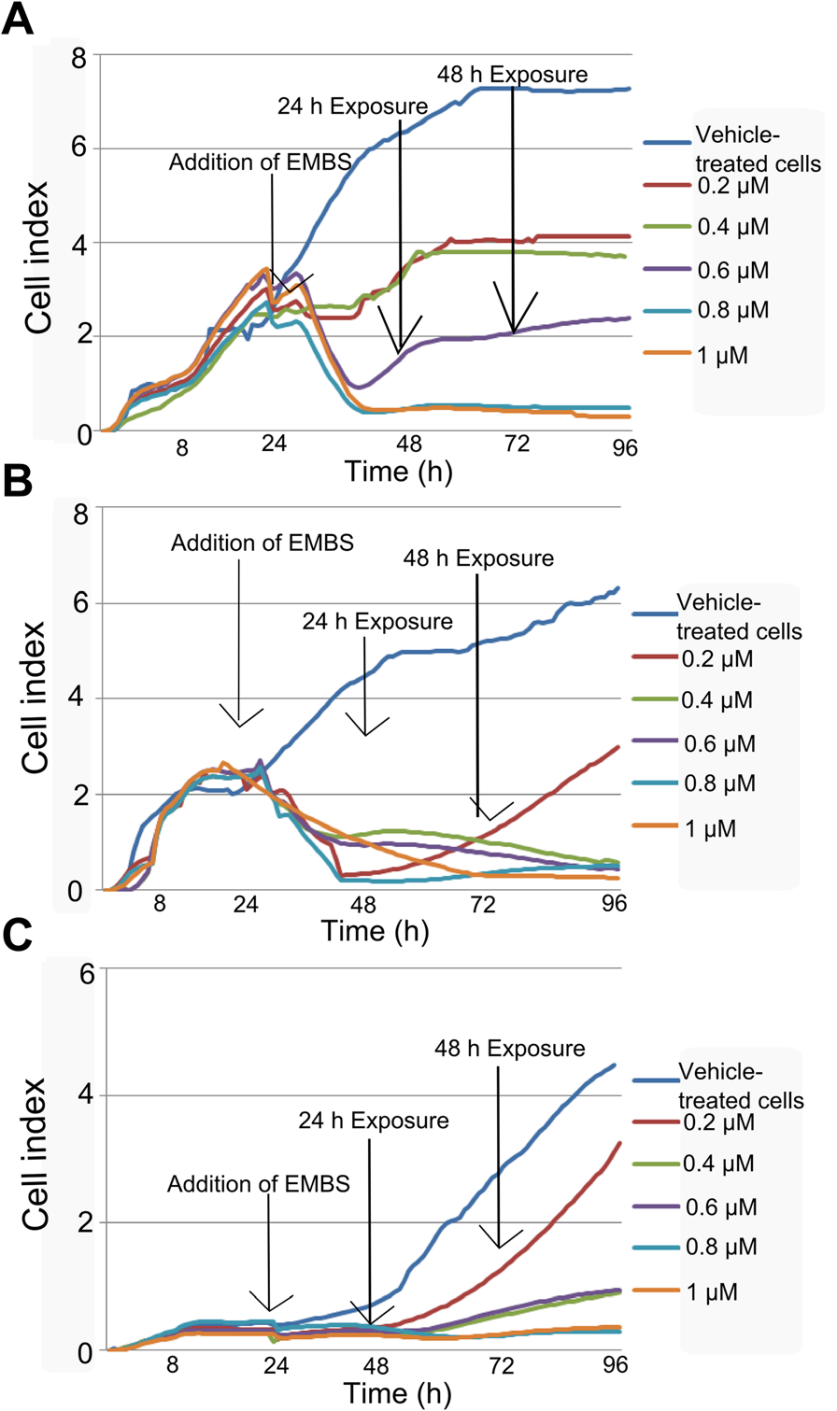
xCELLigence demonstrating effects of EMBS on proliferation. The xCELLigence method demonstrated the in vitro effects of EMBS on proliferation in the MCF-7 **(A)**, MDA-MB-231 **(B)** and MCF-12A cell line **(C)**. Cell proliferation was significantly inhibited at all concentrations in a concentration-dependent manner. However, MCF-12A cell growth recovers after 24 h exposure of EMBS.

A real-time label-independent method was used to measure cell adhesion and cell proliferation after a 24 h exposure of MCF-7, MDA-MB-231 and MCF-12A cells to EMBS (Figure 1). Cell proliferation was inhibited in all 3 cell lines (0.2-1 μM) with decreased cell index observed in a dose-dependent manner. The MCF-7 cell line recovered within 24 h after exposure at the lower doses (0.2-0.6 μM). MDA-MB-231 cell line recovered only after 48 h exposure with less cell proliferation when compared to the vehicle-treated cells. MCF-12A also demonstrated salvaged proliferation after 24 h exposure indicating that the cell line recovered at the lower doses.

### Cell cycle progression

**Figure 2:**
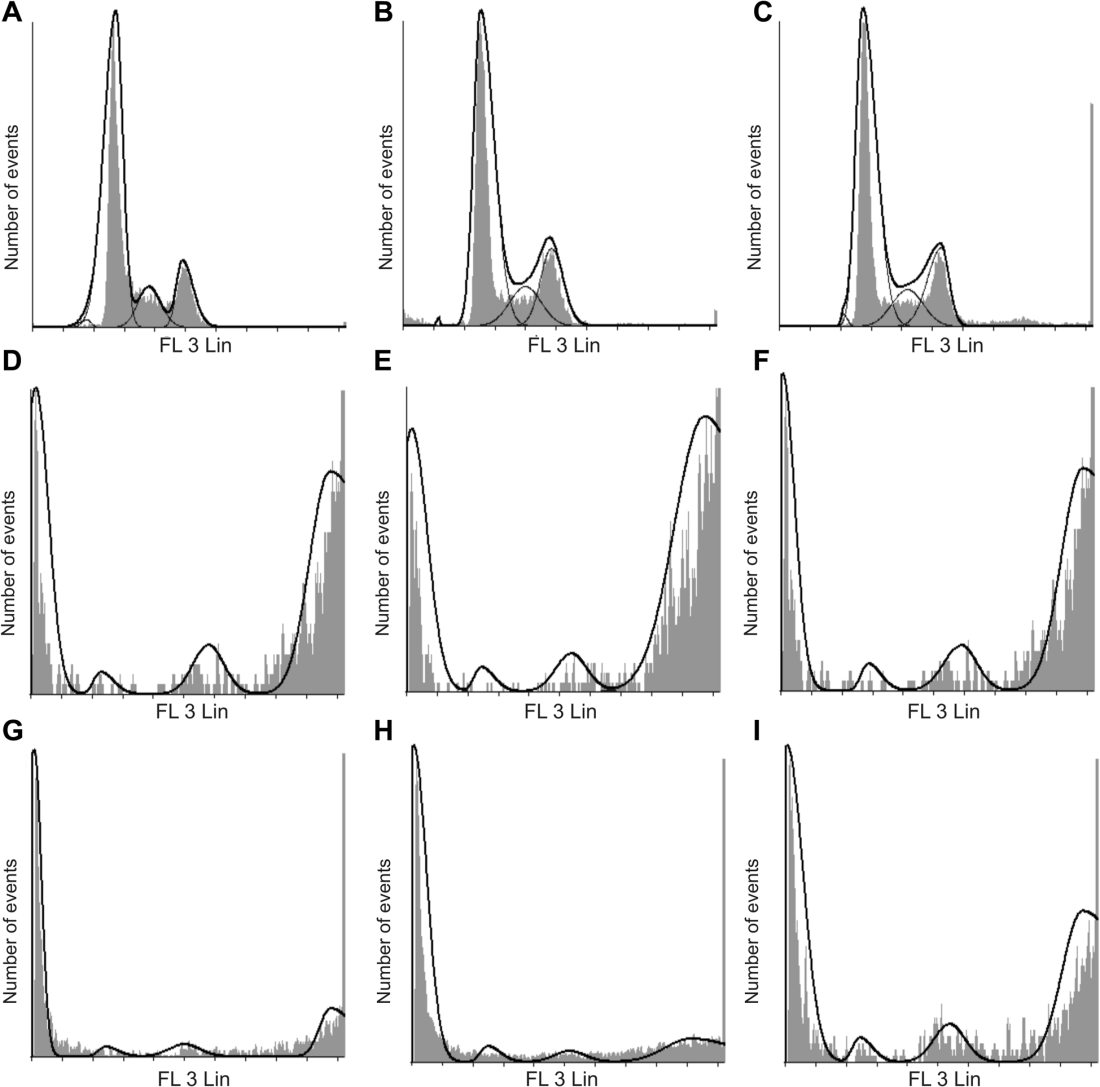
Effects of EMBS on cell cycle progression. Flow cytometrical investigation using propidium iodide and ethanol fixation demonstrated cell cycle progression. Cell cycle progression of vehicle-treated MCF-7 **(A)**, MDA-MB-231 **(B)** and MCF-12A cells **(C)** revealed the majority of cells occupied the G_1_ phase and S phase, less than 2% of cells in the sub-G_1_ fraction, and 13-19% of cells occupying the G_2_M phase. Treated cells were exposed to 0.4 μM for 24 h. EMBS-treated MCF-7 **(D)**, MDA-MB-231 **(E)** and MCF-12A cells **(F)** revealed a statistically significant increased sub-G_1_ peak and a statistically significant increased number of cells occupied the G_2_M phase. Statistical significance was indicated on 3 independent experiments each performed in triplicate with a paired student’s *t*-test. MCF-7 **(G)**, MDA-MB-231 **(H)** and MCF-12A cells **(I)** exposed to 0.1 μg/ml actinomycin D used as a positive control for apoptosis induction resulted in apoptosis induction and an increase of cells occupying the G_2_M phase.

**Figure 3:**
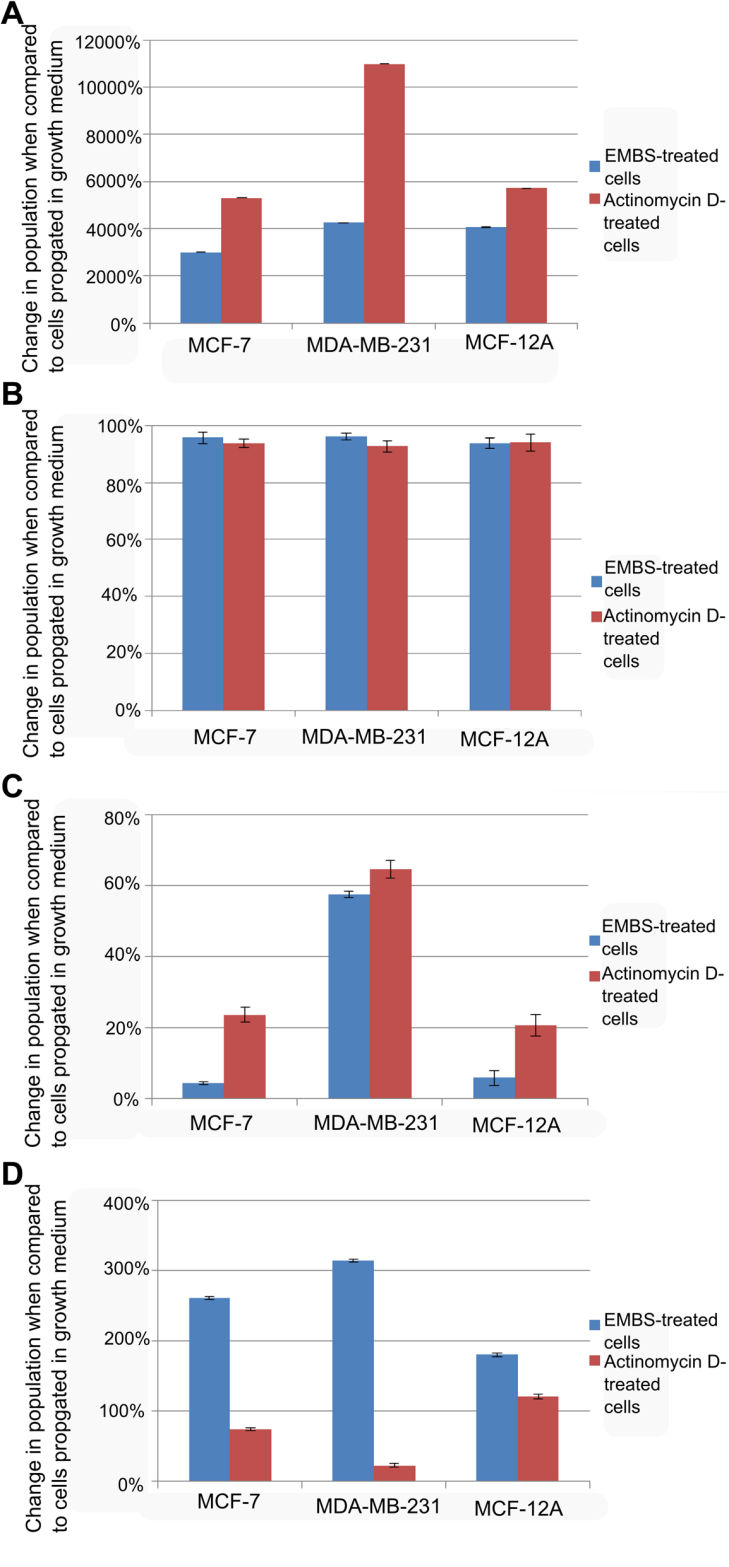
Change in percentage sub-population when compared to cells propagated in growth medium. Percentage change after exposure to EMBS- and actinomycin D in the sub-G_1_ peak **(A)**, G_1_ phase **(B)**, S phase **(C)** and G_2_M phase **(D)**. The percentage change in sub-G_1_ peak was significant after exposure to both EMBS and actinomycin D in all three cell lines. In addition, actinomycin D (positive control) resulted in a large percentage change in all three cell lines. The decrease in cells occupying the G_1_ phase after treatment to EMBS and actinomycin D resulted in a percentage change. The G_2_M phase experienced a large percentage change after exposure to EMBS in all three cell lines most prominently observed in the MDA-MB-231 cell line indicating that EMBS results in mitotic arrest.

Flow cytometry, propidium iodide staining and ethanol fixation was used for the quantification of cell cycle progression, G_2_M block and the presence of apoptosis. Apoptosis was indicated by the existence of a sub-G_1_ phase after exposure to 0.4 μM EMBS for 24 h in MCF-7, MDA-MB-231 and MCF-12A cells (Figures 2, 3 and Table 1). Cell cycle progression after exposure to EMBS resulted in a statistically significant increased sub-G_1_ peak in MCF-7 (36%), MDA-MB-231 (28%) and MCF-12A cells (33%). Furthermore, EMBS exposure also produced a statistically significant increase of MCF-7 (50%), MDA-MB-231(63%) and MCF-12A cells (52%) occupying the G_2_M phase. By contrast, actinomycin D treatment resulted in a general, non cell type specific sub-G1 population, with enrichment in the G_2_M phase, to a lesser extent than for EMBS treatment.

**Table 1:** **Flow cytometry employing propidium iodide and ethanol fixation demonstrating cell cycle progression**

	**Sub G**_**1**_	**G**_**1**_	**S**	**G**_**2**_ **M**
MCF-7 cells				
Vehicle-treated cells	1.16%	73.53%	11.43%	13.86%
EMBS-treated cells	36%*	3.14%	10.92%	50%*
Actinomycin D-treated cells	62.7%*	4.46%	8.72%	24.13%
MDA-MB-231				
Vehicle-treated cells	0.64%	68.95%	15.16%	15.23%
EMBS-treated cells	27.9%*	2.66%	6.45%	63%*
Actinomycin D-treated cells	71%*	4.97%	5.38%	18.69%
MCF-12A cells				
Vehicle-treated cells	0.78%	68.33%	12.42%	18.45%
EMBS-treated cells	32.5%*	4.14%	11.69%	51.7%*
Actinomycin D-treated cells	45.4%*	4.06%	9.85%	40.7%*

A * demonstrates a statistically significant *P* value of <0.05 when compared to vehicle-treated cells.

### Apoptosis induction

**Figure 4:**
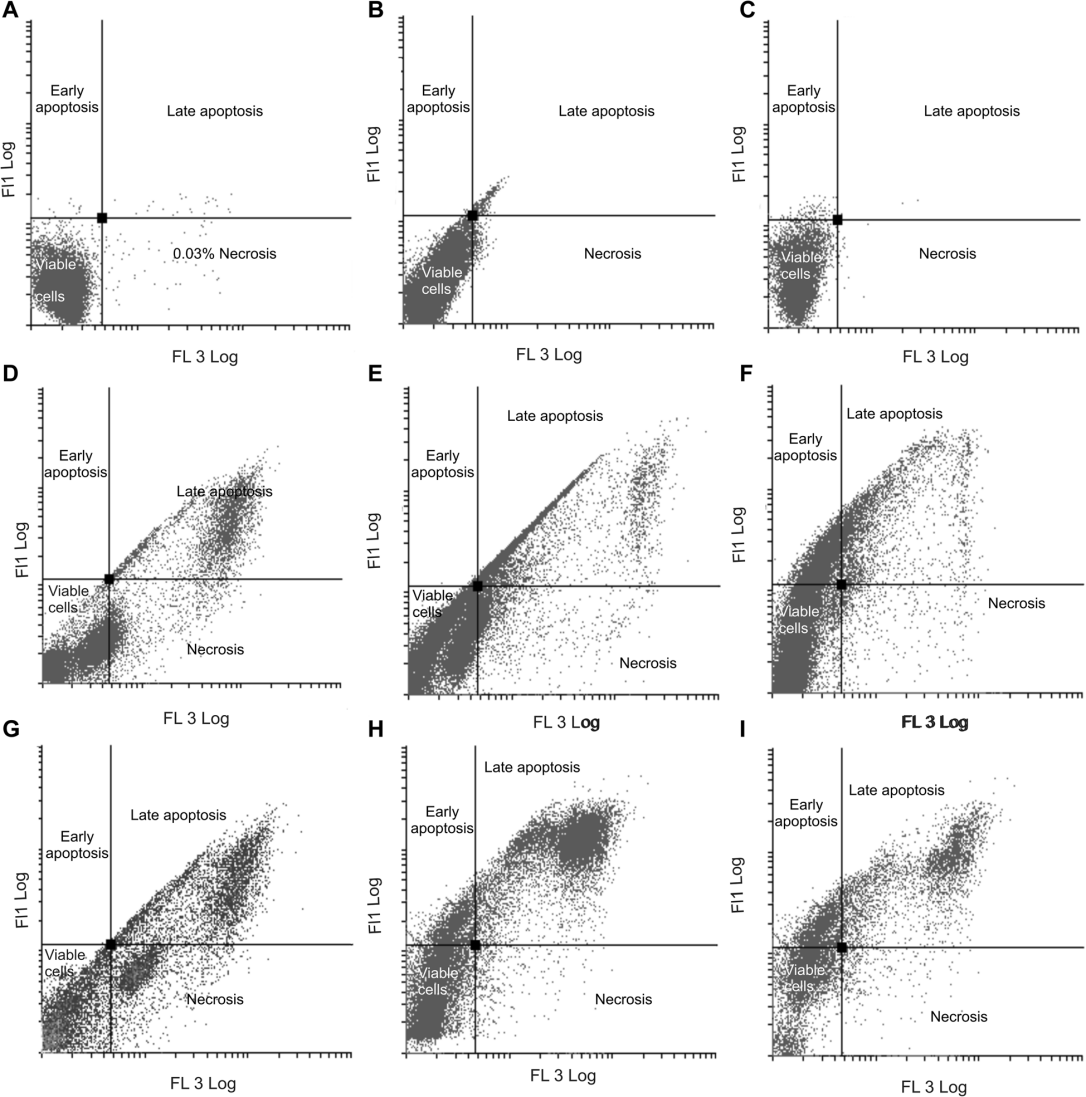
Apoptosis induction by EMBS exposure. Apoptosis induction was also verified using flow cytometry, propidium iodide and annexin V-FITC. Vehicle-treated MCF-7 **(A)**, MDA-MB-231 **(B)** MCF-12A cells **(C)**, EMBS-treated MCF-7 **(D)**, MDA-MB-231 **(E)**, MCF-12 A cells **(F)** and actinomycin D-treated MCF-7 **(G)**, MDA-MB-231 **(H)** and MCF-12A **(I)**. Treated cells demonstrated decreased cell viability accompanied by an increase in cells undergoing late apoptosis and necrosis when compared to vehicle treated cells. Actinomycin D-treated cells used as a positive control for apoptosis induction resulted in decreased cell viability and apoptosis induction. FL3 log (propidium iodide) was represented on the *x*-axis and FL1 log (FITC) was represented on the *y*-axis.

The presence of apoptosis was investigated by means of flow cytometry and Annexin V-FITC (Figure 4 and Table 2). After exposure to EMBS, (0.4 μM, 24 h) MCF-7 cell viability decreased to 66% with 0.1% of cells found present in early apoptosis, 22.21% in late apoptosis and 12.25% in necrosis when compared to vehicle-treated cells. After exposure to EMBS, MDA-MB-231 cell viability decreased to 65% with 0.68% of cells found present in early apoptosis, 21.61% in late apoptosis and 12.31% in necrosis when compared to vehicle-treated cells. After exposure to EMBS, MCF-12A cell viability decreased to 66% with 17% of cells found present in early apoptosis, 11.44% in late apoptosis and 5.11% in necrosis when compared to vehicle-treated cells.

**Table 2:** **Flow cytometry using annexin V-FITC demonstrating viable cells, cells in early apoptosis, cells in late apoptosis and necrotic cells**

	**Viable cells**	**Cells occupying early apoptosis**	**Cells occupying late apoptosis**	**Necrotic cells**
MCF-7 cells				
Vehicle-treated cells	99.3%	0.3%	0.1%	0.3%
EMBS-treated cells	65.5%*	0.0%	22.21%*	12.3%
Actinomycin D-treated cells	49.3%*	0.0%	30.11%*	20.5%*
MDA-MB-231				
Vehicle-treated cells	98.6%	0.0%	0.6%	0.9%
EMBS-treated cells	65.4%*	0.7%	21.6%*	12.3%
Actinomycin D-treated cells	48.4%*	9.3%	40.3%*	1.9%
MCF-12A cells				
Vehicle-treated cells	98.6%	1.1%	0.0%	0.2%
EMBS-treated cells	66.13%*	17.4%	11.4%	5.1%
Actinomycin D-treated cells	46.57%*	32.71%*	17.6%	3.1%

A * demonstrates a statistically significant *P* value of <0.05 when compared to vehicle-treated cells.

### Mitochondrial membrane potential

**Figure 5:**
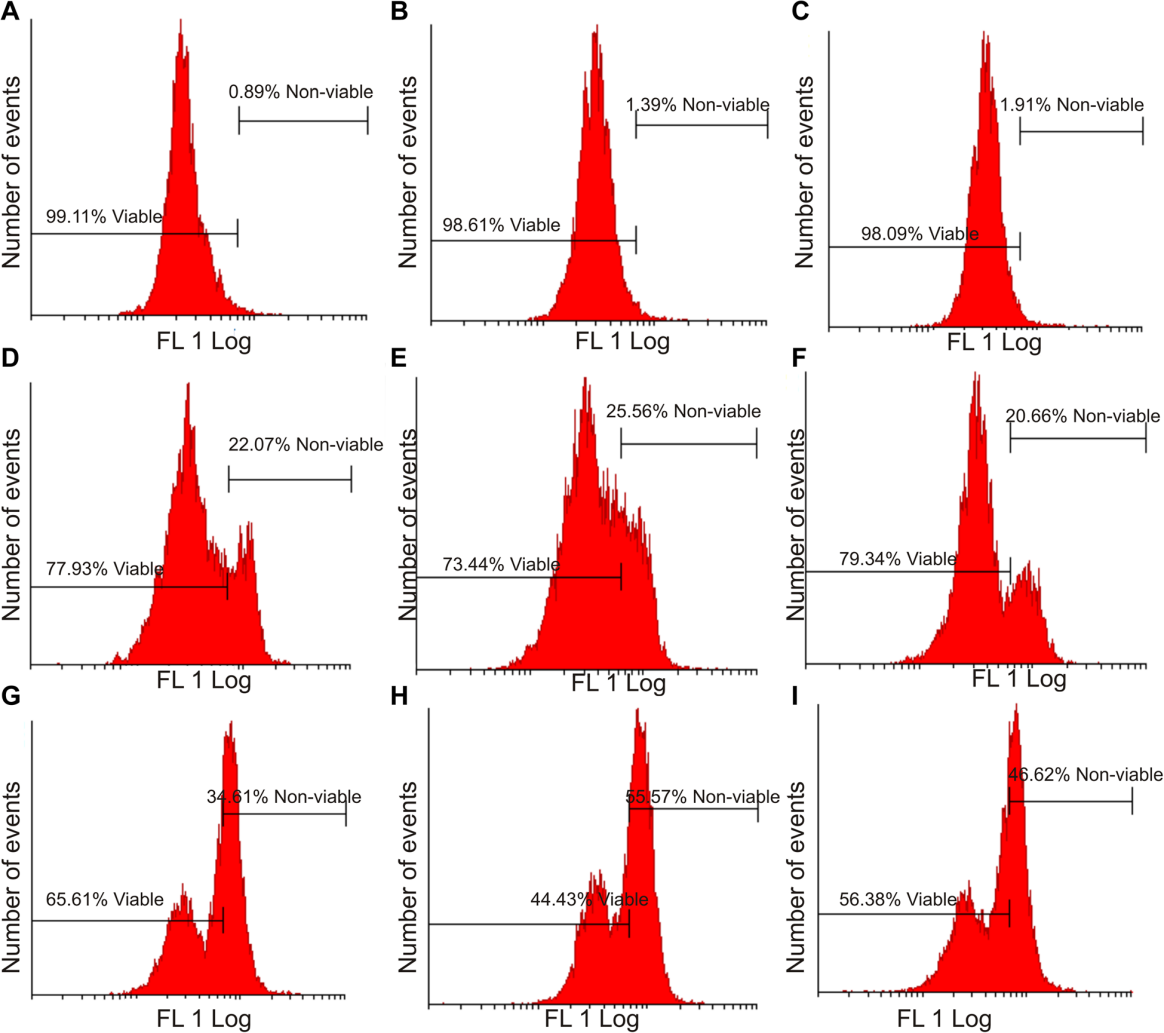
Effects of EMBS on the mitochondrial membrane potential. Mitotracker-stained vehicle-treated MCF-7 **(A)**, MDA-MB-231 **(B)** MCF-12A cells **(C)**, EMBS-treated MCF-7 **(D)**, MDA-MB-231 **(E)**, MCF-12 A cells **(F)** and actinomycin D-treated MCF-7 **(G)**, MDA-MB-231 **(H)** and MCF-12A **(I)**. An increase was observed in the number of cells with reduced mitochondrial potential after treatment with EMBS when compared to the vehicle-treated control.

Further investigation of the intrinsic pathway was performed by demonstrating the effects of EMBS (0.4 μM, 24 h) on the mitochondrial membrane potential (Figure 5). After EMBS exposure 22%, 26% and 21% of MCF-7, MDA-MB-231 and MCF-12A cells possessed mitochondrial depolarization.

### Caspase activation

**Figure 6:**
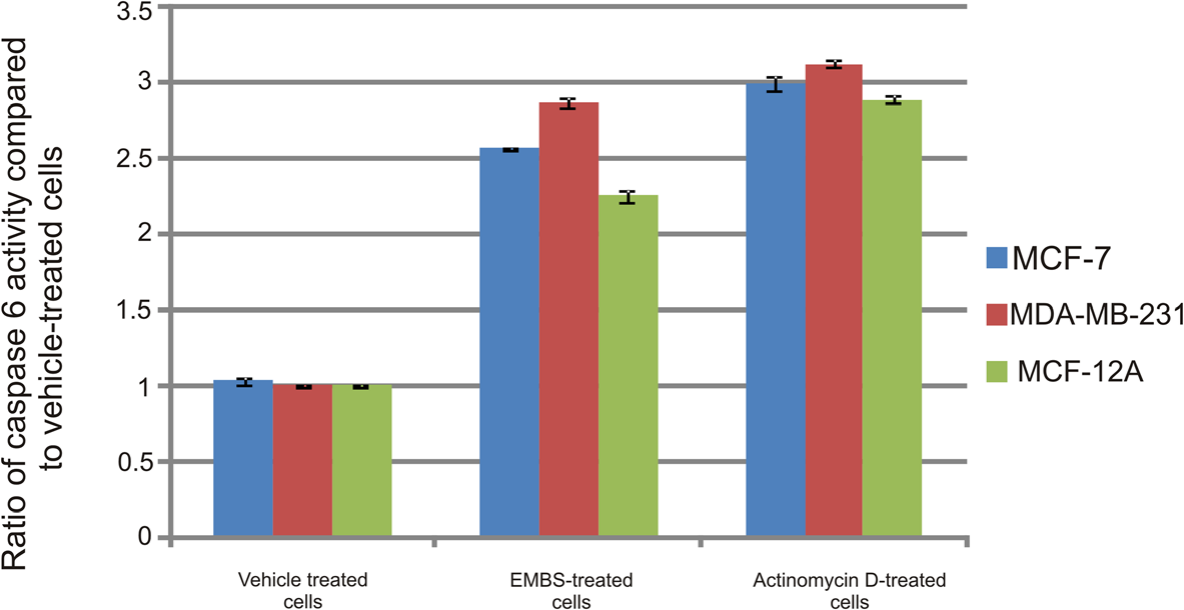
Caspase 6 activation by EMBS. Caspase 6 activity ratios of EMBS- and actinomycin D-treated cells compared to vehicle-treated cells. EMBS-treated MDA-MB-231 cells possessed the highest caspase 6 activity, followed by MCF-7 and MCF-12A. Actinomycin D-treated cells were used as a positive control for apoptosis and caspase activation and displayed significant elevated caspase 6 activity when compared to vehicle-treated cells.

**Figure 7:**
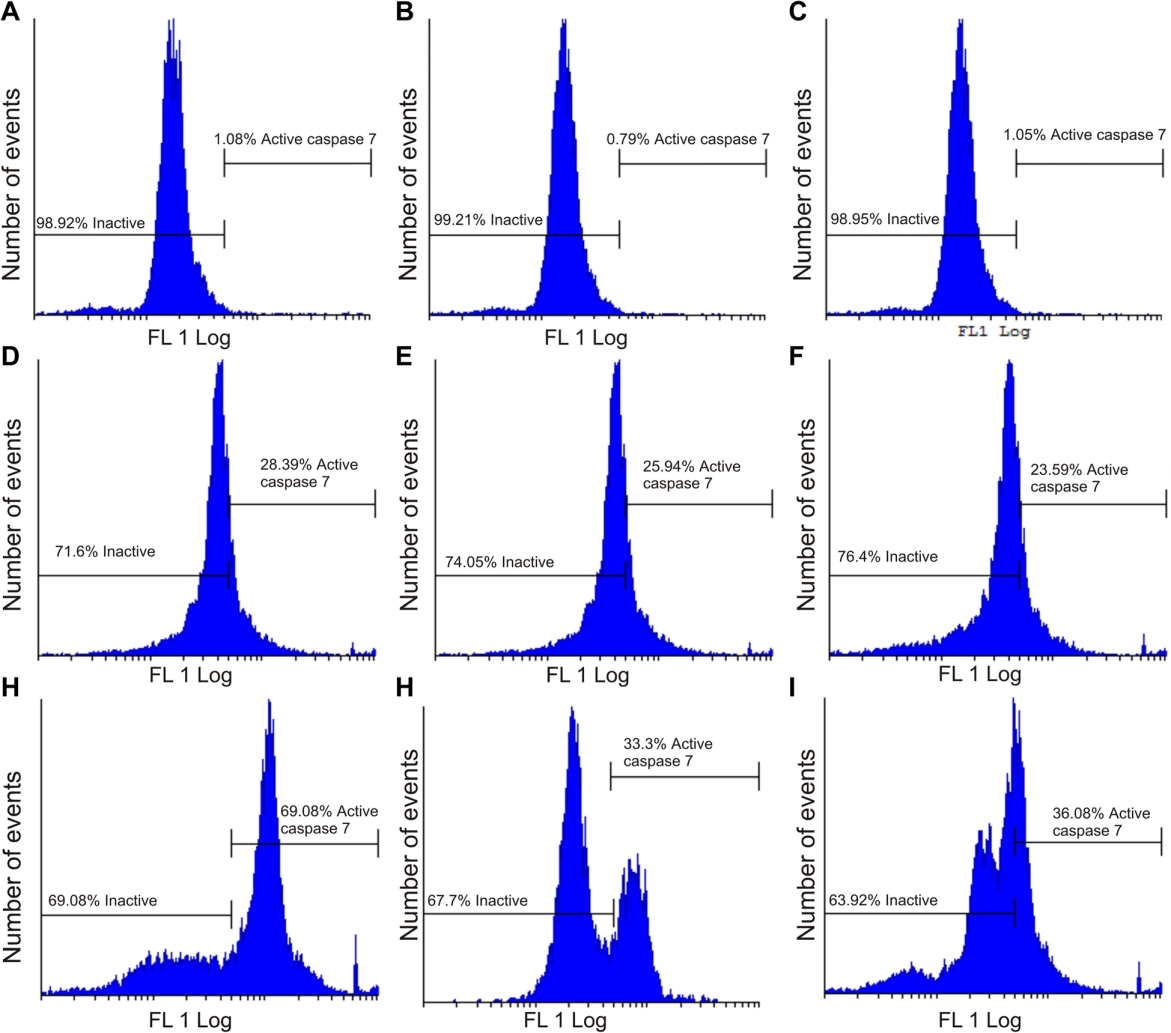
Caspase 7 activation by EMBS. Caspase 7 activity of vehicle-treated MCF-7 **(A)**, MDA-MB-231 **(B)** MCF-12A cells **(C)**, EMBS-treated MCF-7 **(D)**, MDA-MB-231 **(E)**, MCF-12 A cells **(F)** and actinomycin D-treated MCF-7 **(G)**, MDA-MB-231 **(H)** and MCF-12A **(I)**. Data showed an increase in the number of cells with increased caspase 7 activity after exposure to EMBS when compared to cells vehicle-treated cells. Actinomycin D-treated cells were used as a positive control for apoptosis and caspase activation and displayed significant elevated caspase 7 activity when compared to vehicle-treated cells.

**Figure 8:**
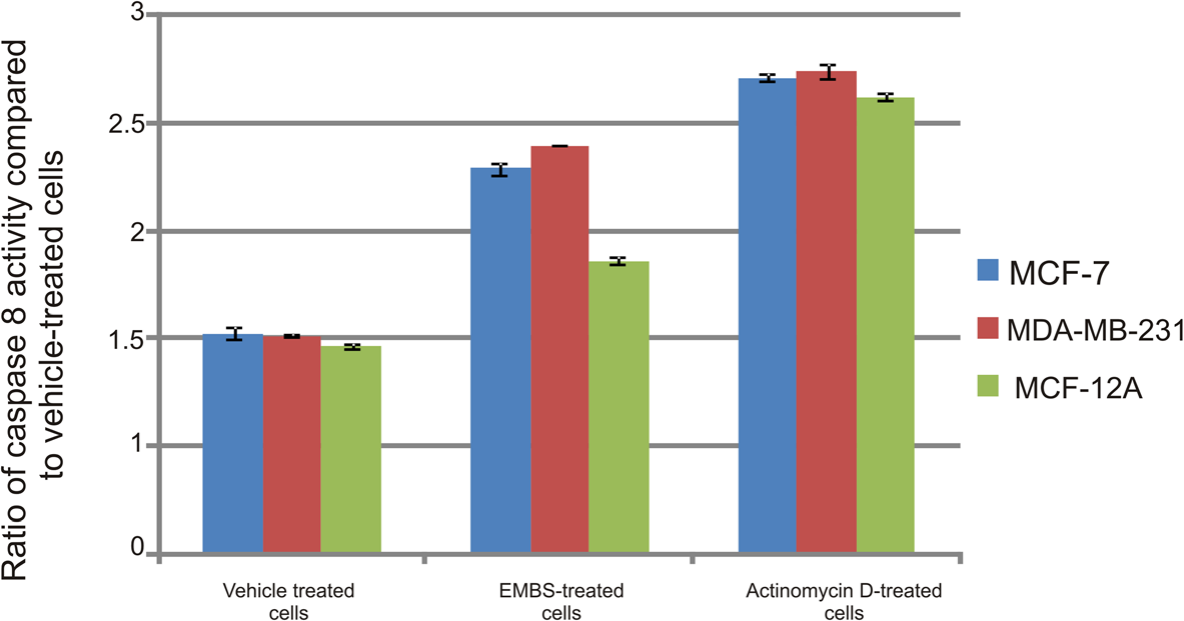
Caspase 8 activation by EMBS. Caspase 8 activity ratios of EMBS- and actinomycin D-treated cells compared to vehicle-treated cells. EMBS-treated MDA-MB-231 cells possessed the highest caspase 8 activity, followed by MCF-7 and MCF-12A. Actinomycin D-treated cells were used as a positive control for apoptosis and caspase activation and displayed significant elevated caspase 8 activity above 2.5 fold when compared to vehicle-treated cells.

Apoptosis induction was also investigated by demonstrating the effect of EMBS (0.4 μM, 24 h) on caspase 6, −7 and −8 activity in MCF-7, MDA-MB-231 and MCF-12A cells lines. Results indicated that EMBS exposure resulted in increased caspase 6-, caspase 7- and caspase 8 activity when compared to vehicle-treated cells. Caspase 6 colorimetric assay revealed that MDA-MB-231 cells were more pronouncedly affected followed by MCF-7 cells when compared to vehicle-treated cells (Figure 6). EMBS-treated cells showed increased caspase 7 activity (23-28%) when compared to vehicle-treated cells (Figure 7). Caspase 8 colorimetric assay also suggested that MDA-MB-231 cells were pronouncedly affected followed by MCF-7 when compared to vehicle-treated cells regarding caspase 8 activity (Figure 8).

### Expression of p53

**Figure 9:**
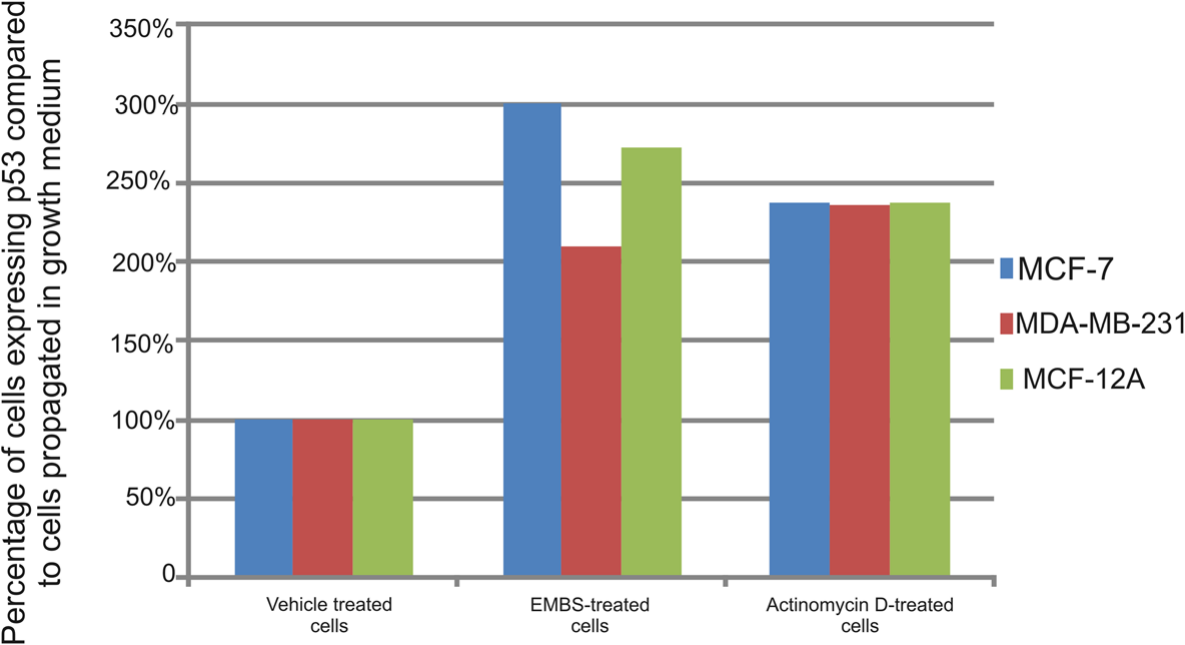
Effects of EMBS on p53 expression. The protein expression of p53 was significantly increased when exposed to 0.4 μM for 24 h. The p53 protein expression of EMBS-treated MCF-7 cells were the most profoundly affected, followed by MCF-12A and MDA-MB-231 when compared to cells propagated in growth medium and vehicle-treated cells.

This study also demonstrated p53 involvement in apoptosis induction by EMBS (0.4 μM, 24 h). Spectrophotometrical data indicated that EMBS exposure resulted in a statistically significant increase of p53 protein expression (Figure 9). The p53 protein expression of MCF-7 cells was the most pronouncedly affected, followed by MDA-MB-231 cells and MCF-12A cells.

### Bcl-2 expression

**Figure 10:**
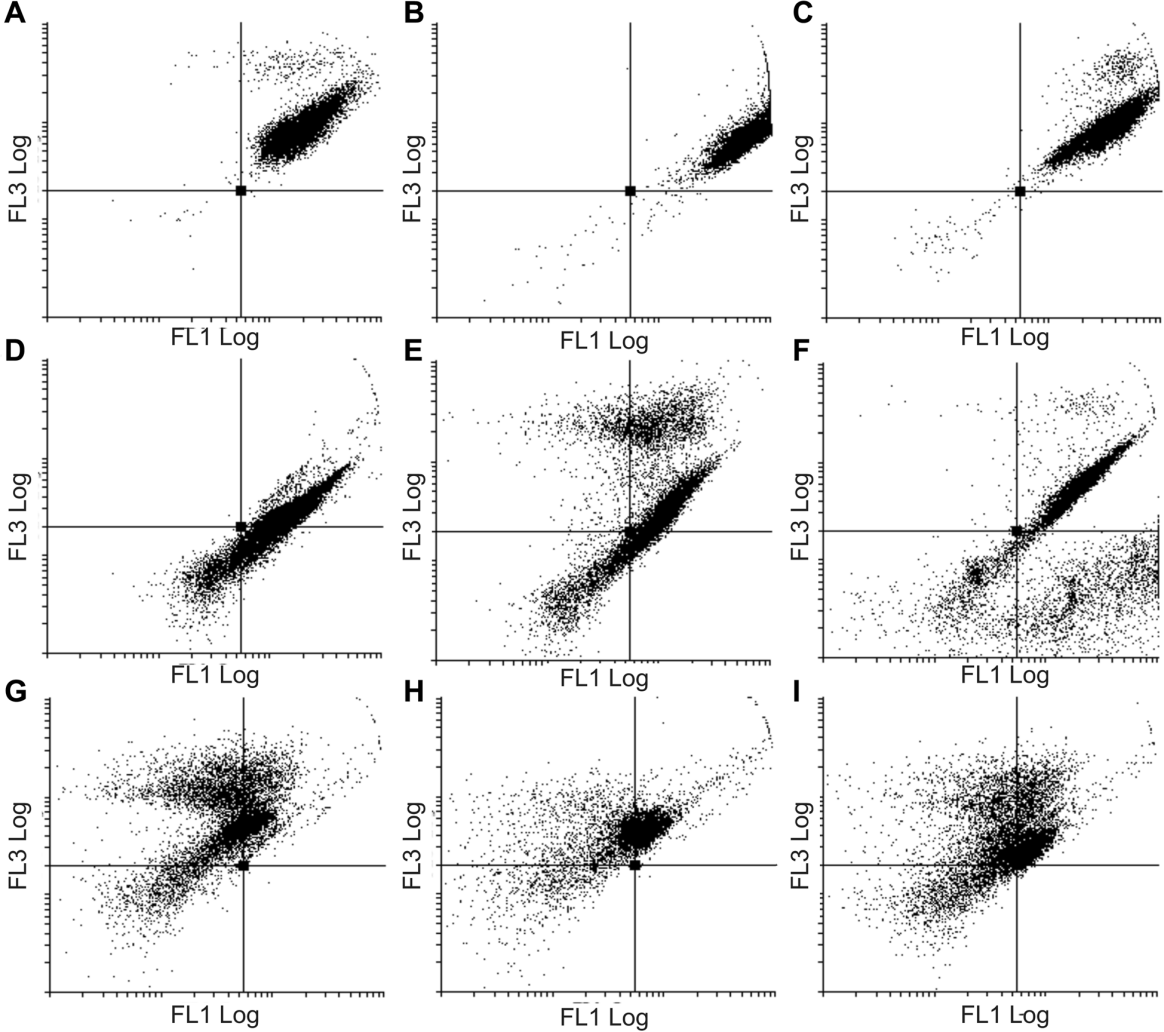
Effects of EMBS on Bcl-2 expression and pBcl-2(s70) phosphorylation. Flow cytometry investigation of Bcl-2 expression (FL1 Log) and pBcl-2(s70) phosphorylation (FL3 Log) of vehicle-treated MCF-7 **(A)**, MDA-MB-231 **(B)** and MCF-12A cells **(C)**. Treated cells were exposed to 0.4 μM for 24 h and demonstrated a downregulated protein expression of Bcl-2 and pBcle-2(s70) phosphorylation status in MCF-7 **(D)**, MDA-MB-231 **(E)** and MCF-12A cells **(F)**. Actinomycin D-treated cells were used as a positive control for apoptosis and showed a decrease in Bcl-2 protein expression and Bcl-2(s70) phosphorylation status in MCF-7 **(G)**, MDA-MB-231 **(H)** and MCF-12A cells **(I)**.

Bcl-2 protein expression and phosphorylation status at Serine 70 of Bcl-2 was investigated using a specialized Flow Collect Bcl-2 activation dual detection kit and flow cytometry. Cells treated with EMBS possessed downregulated Bcl-2 protein expression and a decrease in pBcl-2(s70) phosphorylation status when compared to cells propagated in growth medium and vehicle-treated cells (Figure 10 and Table 3).

**Table 3:** **Data regarding Bcl-2 protein expression and pBcl-2(s70) phosphorylation**

	**Standard Bcl-2 expression and pBcl-2(s70) phosphorylation**	**Down-regulated Bcl-2; standard pBcl-2(s70) phosphorylation**	**Down-regulated Bcl-2 expression and pBcl-2(s70) phosphorylation**	**Standard Bcl-2 expression; down-regulated pBcl-2(s70) phosphorylation**
MCF-7 cells				
Vehicle-treated cells	99.2%	0.6%	0.1%	0.0%
EMBS-treated cells	63.06%*	0.0%	14.2%	22.69%*
Actinomycin D-treated cells	36.53%*	54.38%*	9.0%	0.0%
MDA-MB-231 cells				
Vehicle-treated cells	99.3%	0.0%	0.3%	0.3%
EMBS-treated cells	61.75%*	9.5%	17.4%	11.4%
Actinomycin D-treated cells	27.78%*	65.13%*	7.1%	0.0%
MCF-12A cells				
Vehicle-treated cells	99.1%	0.1%	0.7%	0.0%
EMBS-treated cells	70.14%*	0.6%	10.9%	18.3%
Actinomycin D-treated cells	35.07%*	50.18%*	35.07%*	0.4%

*Statistical significance at *P* <0.05 compared to vehicle-treated cells in a paired student *T*-test.

## Discussion

Microtubule agents have been studied for decades as effective anticancer agents resulting in the *in silico*-design of several estradiol analogues and subsequent synthesis [3]. This in vitro study investigated the influence of a 17-beta estradiol analogue, EMBS, on proliferation, cell cycle progression and apoptosis induction in an adenocarcinoma receptor positive cell line (MCF-7), estrogen receptor-negative metastatic cell line (MDA-MB-231) and a non-tumorigenic epithelial breast cell line (MCF-12A).

The xCELLigence system demonstrated successful antiproliferative activity in an estrogen-independent manner. This was supported by another recent study conducted in our laboratory that reported successful antiproliferation activity exerted by EMBS (0.4 μM; 24 h) in MCF-7, MDA-MB-231 and MCF-12A cell lines [12]. EMBS was previously found to exert antiproliferative activity in cell lines including lung adenocarcinoma cell line (HOP-62), human colorectal carcinoma cell line (HCT-116), human glioblastoma cell line (SF-539), melanoma cell line (UAVC-62), human ovarian adenocarcinoma cell line (OVCAR-3), renal carcinoma cell line (SN12-C), human prostate carcinoma cell line (DU-145) and a breast tumorigenic cell line (MDA-MB-435) [19]. Previous studies have also reported that that other sulphamoylated estradiol compounds including 2-methoxyestradiol-bis-sulphamate, 2-ethyl-3-*O*-sulphamoyl-estra-1,3,5(10)16-tetraene and 2-ethyl-3-*O*-sulphamoyl-estra-1,3,5(10)15-tetraene-3-ol-17one reduced cell growth in a similar concentration range that was observed in this study [9,20,21].

Cell cycle progression studies have revealed that EMBS exposure (0.4 μM; 24 h) results in a statistically significantly increased number of cells occupying the G_2_M phase and subsequent apoptosis induction verified by means of flow cytometry employing annexin V-FITC. Antimitotic activity exerted by EMBS is supported by findings of a previous study that revealed an increase in cells trapped in metaphase resulting in apoptosis [12]. In addition, this study also suggests that EMBS exposure results in apoptosis induction by means of depolarisation of the mitochondrial membrane and caspase 6, −7 and −8 activation in all three cell lines. Previous studies have reported that exposure to 2-ethyl-3-*O*-sulphamoyl-estra-1, 3, 5 (10) 16-tetraene and 2-ethyl-3-*O*-sulphamoyl-estra-1, 3, 5 (10) 15-tetraene-3-ol-17one resulted in increased caspase 6 and caspase 8 activity [21]. In addition, 2-ethyl-3-*O*-sulphamoyl-estra-1, 3, 5 (10) 16-tetraene, 2-ethyl-3-*O*-sulphamoyl-estra-1, 3, 5 (10) 15-tetraene-3-ol-17one reduced the mitochondrial membrane potential in the human cervical adenocarcinoma cell line (HeLa) [21]. The depolarization of the mitochondrial membrane potential suggests apoptosis induction via the intrinsic pathway. EMBS exposure produced depolarization of the mitochondrial membrane potential and cytochrome *c* release in MDA-MB-231 cell line and human umbilical vein endothelial cell line (HUVEC) [22]. The increased quantity of cells possessing reduced mitochondrial membrane potential and increased caspase 8 activity indicates that EMBS induces apoptosis by means of both the intrinsic and extrinsic pathways.

Spectrophotometry results revealed that EMBS-treated cells exhibited increased p53 protein expression. The finding is supported by reports that another sulphamoylated analogue, 2-ethylestrone-3-sulfamate upregulated p53 expression in CAL-51 cell lines, HUVEC cell lines and MCF-7 cells line [23,24]. The p53 tumor suppressor is the primary mediator of proliferation arrest, senescence, and apoptosis induction. In unstressed cells murine double minute 2 (MDM2), the main antagonist of p53, continuously monoubiquitinates p53 and therefore this is the essential step in regulating its degradation by nuclear and cytoplasmic proteosomes [25]. Increased p53 protein expression after exposure to EMBS suggests the presence of decreased MDM2 and Akt expression. P53 targets several pro-apoptotic genes including Bcl-2-Associated X Protein (BAX), Fas/Apo1, Killer/death receptor 5 (DR5), PUMA, Noxa, AFAF1 and Bcl-2 [26]. Gene array studies conducted in our laboratory revealed that BAX expression was increased in the MDA-MB-231 cell line after exposure to EMBS [27]. Transcriptional upregulation by p53 of BAX results in a conformational change and triggers the intrinsic apoptotic pathway [28].

Unregulated p53 expression is supported by the decreased Bcl-2 protein expression and a decrease in pBcl-2(s70) phosphorylation status after exposure to EMBS when compared to vehicle-treated cells. Bcl-2 is an anti-apoptotic protein that occurs on the cytoplasmic face of the mitochondrial outer membrane and endoplasmic reticulum. Bcl-2 alters the cellular organelle’s behaviour by modifying the flux of small molecules or proteins [29]. Bcl-2 protects against apoptosis by stabilization the mitochondrial membrane potential. This stabilization prevents the release of cytochrome *c* and activation of caspase 2 and caspase 9 [30]. Thus this study suggests that apoptosis induction is also mediated by decreased p53- and Bcl-2 protein expression accompanied by decreased pBcl-2(s70) phosphorylation status.

## Conclusion

In conclusion, the aims of this study namely, to investigate the effects of EMBS in breast adenocarcinoma cell lines (MCF-7 and MDA-MB-231) and the non-tumorigenic epithelial breast cell line (MCF-12A) respectively by analyzing its influence on cell growth, cell cycle progression and apoptosis induction were achieved. EMBS exposure resulted in antiproliferative-and antimitotic activity and induced apoptosis in an estrogen receptor-independent manner. The induction of both the intrinsic- and extrinsic apoptotic pathway by EMBS exposure is implicated. Data collected in this study indicate that EMBS induces intrinsic apoptosis by means of a reduction in the mitochondrial membrane potential that results in the activation of caspases 6- and −7 accompanied with a decrease in Bcl-2 protein expression and pBcl-2(s70) phosphosphorylation status. The involvement of the extrinsic apoptotic pathway is also indicated by the activation of caspase 8. EMBS exposure resulted in increased caspase 6-, caspase 7- and caspase 8 activity, upregulated p53 protein expression and a decrease in phosphorylation status of Bcl-2 at serine 70 in tumorigenic and non-tumorigenic lines. Data contribute to the unravelling of the signalling mechanism utilized by EMBS in breast tumorigenic and non-tumorigenic cell lines and to the development and/or improvement of novel chemotherapeutic agents paving the way for efficient in vivo studies.
